# Poly[di-μ_2_-aqua-μ_2_-(5-methyl­pyrazine-2-carboxyl­ato)-(5-methyl­pyrazine-2-carboxyl­ato)-μ_3_-nitrato-trilithium]

**DOI:** 10.1107/S1600536811024548

**Published:** 2011-06-30

**Authors:** Wojciech Starosta, Janusz Leciejewicz

**Affiliations:** a Institute of Nuclear Chemistry and Technology, ul.Dorodna 16, 03-195 Warszawa, Poland

## Abstract

The asymmetric unit of the title compound, [Li_3_(C_6_H_5_N_2_O_2_)_2_(NO_3_)(H_2_O)_2_]_*n*_ contains three Li^I^ ions, two ligand anions, two water mol­ecules and a nitrate anion. Related by a centre of inversion, they form a centrosymmetric mol­ecular cluster in which one of the Li^I^ ions shows trigonal–bipyramidal and the other two distorted tetra­hedral coordination. Li^I^ ions are bridged by water O atoms and carboxyl­ate O atoms donated by one of the ligands. The clusters, bridged by two nitrato O atoms, form mol­ecular columns along [010], which are held together by O—H⋯O and O—H⋯N hydrogen bonds and π–π inter­actions [centroid–centroid distances = 3.694 (1) and 3.796 (1) Å].

## Related literature

For the structure of a lithium complex with 3-amino­pyrazine-2-carboxyl­ate and aqua ligands, see: Starosta & Leciejewicz (2010 ▶). The structures of two complexes with pyridazine carboxyl­ate ligands have been also determined, see: Starosta & Leciejewicz (2011*a* ▶,*b* ▶). For the structure of a Li^I^ complex with pyrimidine carboxyl­ate and nitrate ligands, see: Starosta & Leciejewicz (2011*c* ▶).
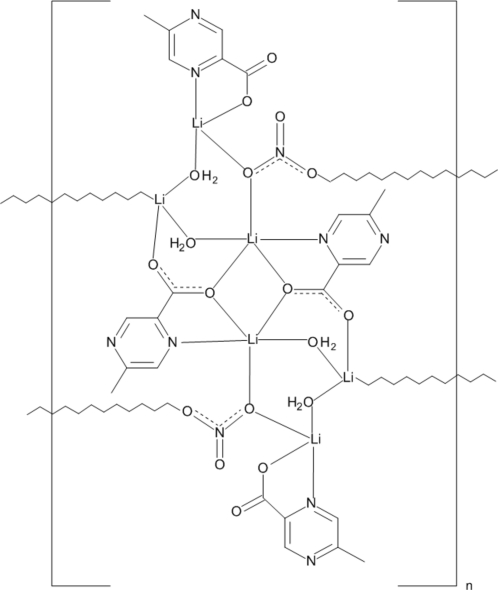


## Experimental

### 

#### Crystal data

[Li_3_(C_6_H_5_N_2_O_2_)_2_(NO_3_)(H_2_O)_2_]
*M* *_r_* = 393.10
Monoclinic, 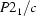

*a* = 13.0222 (1) Å
*b* = 7.2288 (1) Å
*c* = 18.5819 (2) Å
β = 100.760 (1)°
*V* = 1718.45 (3) Å^3^
*Z* = 4
Cu *K*α radiation
μ = 1.10 mm^−1^
*T* = 293 K
0.23 × 0.20 × 0.07 mm

#### Data collection

Oxford Diffraction Xcalibur Ruby diffractometer
Absorption correction: multi-scan (*CrysAlis PRO*; Oxford Diffraction, 2010 ▶) *T*_min_ = 0.672, *T*_max_ = 1.000
15696 measured reflections
3215 independent reflections
2787 reflections with *I* > 2σ(*I*)
*R*_int_ = 0.026

#### Refinement

*R*[*F* ^2^ > 2σ(*F* ^2^)] = 0.040
*wR*(*F* ^2^) = 0.122
*S* = 1.07
3215 reflections
277 parameters
H atoms treated by a mixture of independent and constrained refinement
Δρ_max_ = 0.35 e Å^−3^
Δρ_min_ = −0.31 e Å^−3^

### 

Data collection: *CrysAlis PRO* (Oxford Diffraction, 2010 ▶); cell refinement: *CrysAlis PRO*; data reduction: *CrysAlis PRO*; program(s) used to solve structure: *SHELXS97* (Sheldrick, 2008 ▶); program(s) used to refine structure: *SHELXL97* (Sheldrick, 2008 ▶); molecular graphics: *SHELXTL* (Sheldrick, 2008 ▶); software used to prepare material for publication: *SHELXTL*.

## Supplementary Material

Crystal structure: contains datablock(s) I, global. DOI: 10.1107/S1600536811024548/kp2334sup1.cif

Structure factors: contains datablock(s) I. DOI: 10.1107/S1600536811024548/kp2334Isup2.hkl

Additional supplementary materials:  crystallographic information; 3D view; checkCIF report

## Figures and Tables

**Table 1:** Selected bond lengths (Å)

Li1—O11^i^	2.029 (3)
Li1—O11	2.039 (3)
Li1—O1	2.085 (3)
Li1—O4	2.114 (3)
Li1—N11	2.293 (3)
Li2—O12^i^	1.958 (3)
Li2—O4	1.965 (3)
Li2—O5	1.970 (3)
Li2—O2^ii^	2.163 (3)
Li3—O31	1.978 (3)
Li3—O5	2.025 (4)
Li3—O1	2.039 (3)
Li3—N31	2.117 (3)

Symmetry codes: (i) 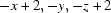; (ii) 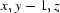.

**Table 2:** Hydrogen-bond geometry (Å, °)

*D*—H⋯*A*	*D*—H	H⋯*A*	*D*⋯*A*	*D*—H⋯*A*
O4—H41⋯O32^iii^	0.85 (2)	1.92 (2)	2.7449 (15)	165.3 (19)
O4—H42⋯N12^iv^	0.85 (2)	2.03 (2)	2.8414 (17)	159.0 (19)
O5—H52⋯N32^v^	0.84 (2)	2.05 (2)	2.8550 (17)	162 (2)
O5—H51⋯O31^iii^	0.86 (2)	1.85 (2)	2.7055 (15)	172 (2)

Symmetry codes: (iii) 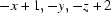; (iv) 
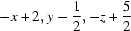
; (v) 
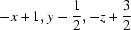
.

**Fig. 1.**
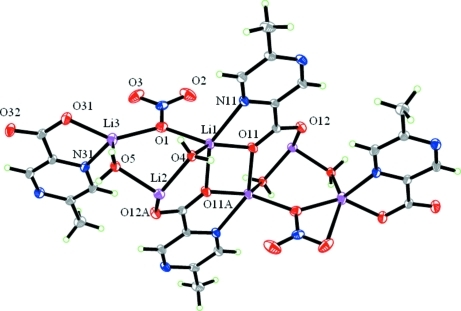
The dimeric structural unit of the title compound with atom labelling scheme and 50% probability displacement ellipsoids. Symmetry code: (i) -x + 2, -y, -z + 2; (ii) x, y - 1, z; (iii) x, y + 1, z.

**Fig. 2.**
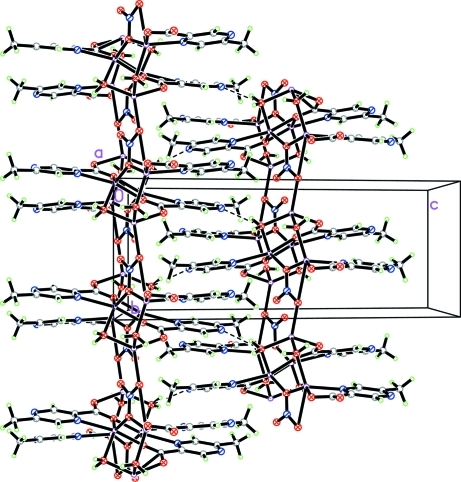
The alignment of the polyhedra columns in the unit cell.

## supplementary crystallographic information

### Comment

The asymmetric unit of the title compound contains three Li^I^ ions, two 5-methylpyrazine-2-carboxylate anions, two water molecules and a nitrate anion (Fig. 1). The coordination environment of the Li1 ion is composed of N11, O11^i^, O1, O4 and O11 atoms. The latter three form a base of a distorted trigonal bipyramid, N11,O11^i^ atoms are at its apices. Li1 ion is 0.0097 (2)Å out of the basal plane. The Li2 ion is coordinated by water O4, O5, carboxylate O12^i^ and nitrate O2^iii^ atoms which form a distorted tetrahedral coordination environment. The same distorted tetrahedral coordination geometry shows the Li3 ion sorrounded by N31, O31, O1 and O5 atoms. The observed Li—O and Li—N bond distances (Table 1) are typical of Li complexes with azine carboxylate ligands. Both methylpyrazine rings are planar with r.m.s. of 0.0074 (1)Å for ring 1 and 0.0069 (1)Å for ring 3; carboxylate groups C17/O11/O12 and C37/O31/O32 make with relevant rings dihedral angles of 11.2 (1)°, and 11,0(1)°, respectively. The nitrate anion is planar [r.m.s. 0.0002 (1) Å]. Its O1 atom acts as bidentate and bridges Li1 and Li3 ions, while the O2 atom chelates the Li2 ion. Nitrato O3 atom is not coordinated at all. Li1 and Li1^i^ ions bridged by bidentate carboxylate O11 and O11^i^ form a core of a centrosymmetric cluster composed of Li1 and Li3 ions bridged by bidentate nitrato O1 atom, Li1 and Li2 bridged by the aqua O4 atom, Li2 and Li3 bridged by the aqua O5 atom. The clusters bridged via nitrato O1 and O2 atoms, form molecular columns along the direction [010] and they are held together by a network of hydrogen bonds in which aqua O4 and O5 molecules are as donors and carboxylate O31 and O32 atoms are acceptors. π-π  interactions between methylpyrazine rings of adjacent columns are defined: the centres of gravity of the ring (N11, C12, C13, N12, C15, C16) and its symmetry generated hetrerocyclic rings [2-x, -1/2+y,5/2-z; 2-x,1/2+y,5/2-z] both are separated by 3.694 (1) Å, and the ring (N31, C32, C33,  N32, C35, C36) operated by symmetry [1-x, -1/2+y, 3/2-z; 1-x, 1/2+y, 3/2-z] generates two equal stacking contacts of 3.796 (1) Å (Fig. 2). However, their shifts are about 3.5 Å. Molecular columns composed of centrosymmetric dimers have been also observed in the structure of a Li^I^ complex with 3-aminopyrazine-2-carboxylate and water ligands (Starosta & Leciejewicz, 2010). Molecular layers built of centrosymmetric dimers have been reported in the structure of a complex with pyridazine-4-carboxylate and water ligands (Starosta & Leciejewicz, 2011a) while centrosymmetric molecular ribbons bridged by nitrate ions form double-layers in the structure of a complex with pyrimidine-2-carboxylate and nitrate ligands (Starosta & Leciejewicz, 2011b). On the other hand, monomeric molecules, in which a Li^I^ ion is chelated by ligand N,O bonding group and two aqua O atoms constitute the structure of a complex with pyridazine-3-carboxylate and water ligands (Starosta & Leciejewicz, 2011a).

### Experimental

Hot aqueous solutions, one containig 1 mmol of 5-methylpyrazine-2-carboxylic acid (Aldrich), the other 1 mmol of lithium (I) nitrate were mixed and boiled under reflux with constant stirring for 5 h. Left for evaporation at room temperature. After a couple of days yellow single-crystal plates of the title complex deposited on the bottom of a crystallization pot. Crystals were washed with cold ethanol and dried in air.

### Refinement

Pyrazine ring H atoms atoms were placed in calculated positions with C—H = 0.93 and 0.96Å and treated as riding on the parent atoms with *U*_iso_(H)= 1.2*U*_eq_(C)or *U*_iso_(H)=1.5*U*_eq_(C_methyl_). Water H atoms were found in Fourier map and refined isotropically.

### Crystal data

**Table:**

[Li_3_(C_6_H_5_N_2_O_2_)_2_(NO_3_)(H_2_O)_2_]	*Z* = 4
*M**_r_* = 393.10	*F*(000) = 808
Monoclinic, *P*2_1_/*c*	*D*_x_ = 1.519 Mg m^−^^3^
Hall symbol: -P 2ybc	Cu *K*α radiation, λ = 1.54184 Å
*a* = 13.0222 (1) Å	µ = 1.10 mm^−^^1^
*b* = 7.2288 (1) Å	*T* = 293 K
*c* = 18.5819 (2) Å	Plate, yellow
β = 100.760 (1)°	0.23 × 0.20 × 0.07 mm
*V* = 1718.45 (3) Å^3^	

### Data collection

**Table:**

Oxford Diffraction Xcalibur Ruby diffractometer	3215 independent reflections
Radiation source: Enhance (Cu) X-ray Source	2787 reflections with *I* > 2σ(*I*)
graphite	*R*_int_ = 0.026
Detector resolution: 10.4922 pixels mm^-1^	θ_max_ = 70.1°, θ_min_ = 3.5°
ω scans	*h* = −15→15
Absorption correction: multi-scan (*CrysAlis PRO*; Oxford Diffraction, 2010)	*k* = −8→7
*T*_min_ = 0.672, *T*_max_ = 1.000	*l* = −22→22
15696 measured reflections	

### Refinement

**Table:**

Refinement on *F*^2^	Primary atom site location: structure-invariant direct methods
Least-squares matrix: full	Secondary atom site location: difference Fourier map
*R*[*F*^2^ > 2σ(*F*^2^)] = 0.040	Hydrogen site location: inferred from neighbouring sites
*wR*(*F*^2^) = 0.122	H atoms treated by a mixture of independent and constrained refinement
*S* = 1.07	*w* = 1/[σ^2^(*F*_o_^2^) + (0.0709*P*)^2^ + 0.484*P*] where *P* = (*F*_o_^2^ + 2*F*_c_^2^)/3
3215 reflections	(Δ/σ)_max_ = 0.001
277 parameters	Δρ_max_ = 0.35 e Å^−^^3^
0 restraints	Δρ_min_ = −0.31 e Å^−^^3^

### Special details

**Table:**

Experimental. (CrysAlis PRO; Oxford Diffraction Ltd., Version 1.171.33.66 (release 28-04-2010 CrysAlis171 .NET) (compiled Apr 28 2010,14:27:37) Empirical absorption correction using spherical harmonics, implemented in SCALE3 ABSPACK scaling algorithm.
Geometry. All e.s.d.'s (except the e.s.d. in the dihedral angle between two l.s. planes) are estimated using the full covariance matrix. The cell e.s.d.'s are taken into account individually in the estimation of e.s.d.'s in distances, angles and torsion angles; correlations between e.s.d.'s in cell parameters are only used when they are defined by crystal symmetry. An approximate (isotropic) treatment of cell e.s.d.'s is used for estimating e.s.d.'s involving l.s. planes.
Refinement. Refinement of *F*^2^ against ALL reflections. The weighted *R*-factor *wR* and goodness of fit *S* are based on *F*^2^, conventional *R*-factors *R* are based on *F*, with *F* set to zero for negative *F*^2^. The threshold expression of *F*^2^ > σ(*F*^2^) is used only for calculating *R*-factors(gt) *etc*. and is not relevant to the choice of reflections for refinement. *R*-factors based on *F*^2^ are statistically about twice as large as those based on *F*, and *R*- factors based on ALL data will be even larger.

### Fractional atomic coordinates and isotropic or equivalent isotropic displacement parameters (Å^2^)

**Table:**

	*x*	*y*	*z*	*U*_iso_*/*U*_eq_	
Li1	0.90455 (19)	0.0331 (4)	1.03878 (13)	0.0379 (6)	
Li2	0.7564 (2)	−0.2877 (4)	0.97211 (14)	0.0373 (6)	
Li3	0.6256 (2)	0.0936 (5)	0.92945 (15)	0.0486 (7)	
O31	0.47521 (9)	0.15187 (19)	0.91969 (6)	0.0482 (3)	
O32	0.32570 (9)	0.1336 (2)	0.83857 (6)	0.0519 (4)	
N31	0.58804 (10)	0.1142 (2)	0.81387 (7)	0.0371 (3)	
C32	0.48413 (11)	0.1347 (2)	0.79451 (8)	0.0324 (3)	
C33	0.43759 (12)	0.1538 (2)	0.72158 (8)	0.0392 (4)	
H33	0.3653	0.1662	0.7095	0.047*	
N32	0.49310 (11)	0.1548 (2)	0.66817 (7)	0.0419 (3)	
C35	0.59618 (13)	0.1305 (2)	0.68670 (9)	0.0378 (4)	
C36	0.64232 (12)	0.1096 (2)	0.76008 (9)	0.0407 (4)	
H36	0.7142	0.0918	0.7720	0.049*	
C37	0.42190 (12)	0.1403 (2)	0.85605 (8)	0.0357 (4)	
C38	0.65821 (16)	0.1281 (3)	0.62641 (10)	0.0541 (5)	
H38A	0.7279	0.0859	0.6454	0.081*	
H38B	0.6609	0.2506	0.6070	0.081*	
H38C	0.6256	0.0461	0.5882	0.081*	
O12	1.20280 (8)	0.20920 (18)	1.11942 (6)	0.0430 (3)	
O11	1.05909 (8)	0.09788 (18)	1.04975 (6)	0.0440 (3)	
N11	0.95349 (10)	0.11349 (19)	1.15973 (7)	0.0353 (3)	
N1	0.77531 (11)	0.3393 (2)	0.98615 (7)	0.0428 (4)	
O1	0.76826 (9)	0.16678 (16)	0.98797 (6)	0.0457 (3)	
O2	0.70264 (13)	0.4302 (2)	0.95323 (9)	0.0748 (5)	
O3	0.85484 (14)	0.4171 (3)	1.01733 (9)	0.0840 (6)	
O5	0.61725 (8)	−0.17265 (17)	0.96171 (6)	0.0378 (3)	
H51	0.5869 (17)	−0.177 (3)	0.9989 (13)	0.057*	
H52	0.5778 (18)	−0.236 (3)	0.9301 (13)	0.057*	
O4	0.81382 (9)	−0.18568 (16)	1.06931 (5)	0.0335 (3)	
C15	0.95823 (13)	0.1348 (2)	1.28881 (8)	0.0373 (4)	
C12	1.05656 (11)	0.1468 (2)	1.17399 (7)	0.0295 (3)	
C16	0.90570 (12)	0.1100 (2)	1.21690 (9)	0.0401 (4)	
H16	0.8339	0.0900	1.2086	0.048*	
C13	1.10941 (12)	0.1720 (2)	1.24494 (8)	0.0360 (4)	
H13	1.1809	0.1954	1.2531	0.043*	
C17	1.11144 (11)	0.1522 (2)	1.10905 (7)	0.0302 (3)	
N12	1.06118 (11)	0.1639 (2)	1.30230 (7)	0.0402 (3)	
C18	0.90366 (16)	0.1308 (3)	1.35290 (10)	0.0538 (5)	
H18A	0.9461	0.0659	1.3928	0.081*	
H18B	0.8377	0.0688	1.3393	0.081*	
H18C	0.8922	0.2551	1.3678	0.081*	
H42	0.8562 (17)	−0.250 (3)	1.0999 (12)	0.050 (5)*	
H41	0.7640 (17)	−0.161 (3)	1.0912 (11)	0.048 (5)*	

### Atomic displacement parameters (Å^2^)

**Table:**

	*U*^11^	*U*^22^	*U*^33^	*U*^12^	*U*^13^	*U*^23^
Li1	0.0343 (12)	0.0511 (16)	0.0284 (11)	−0.0012 (11)	0.0061 (9)	−0.0045 (11)
Li2	0.0387 (13)	0.0443 (15)	0.0308 (12)	−0.0021 (11)	0.0115 (10)	−0.0018 (11)
Li3	0.0405 (14)	0.073 (2)	0.0317 (13)	0.0052 (14)	0.0061 (11)	0.0076 (13)
O31	0.0377 (6)	0.0820 (9)	0.0270 (5)	0.0121 (6)	0.0111 (4)	0.0055 (5)
O32	0.0318 (6)	0.0881 (10)	0.0383 (6)	0.0059 (6)	0.0127 (5)	0.0048 (6)
N31	0.0315 (6)	0.0520 (8)	0.0286 (6)	0.0081 (6)	0.0075 (5)	0.0056 (5)
C32	0.0307 (7)	0.0395 (8)	0.0280 (7)	0.0055 (6)	0.0078 (6)	0.0043 (6)
C33	0.0318 (8)	0.0559 (10)	0.0300 (7)	0.0047 (7)	0.0061 (6)	0.0059 (7)
N32	0.0425 (8)	0.0568 (9)	0.0271 (6)	0.0027 (6)	0.0081 (5)	0.0045 (6)
C35	0.0411 (8)	0.0423 (9)	0.0327 (7)	0.0025 (7)	0.0143 (6)	0.0017 (6)
C36	0.0311 (7)	0.0565 (10)	0.0366 (8)	0.0083 (7)	0.0120 (6)	0.0049 (7)
C37	0.0332 (8)	0.0464 (9)	0.0292 (7)	0.0086 (6)	0.0102 (6)	0.0063 (6)
C38	0.0568 (11)	0.0709 (13)	0.0409 (9)	0.0036 (9)	0.0256 (8)	0.0030 (9)
O12	0.0359 (6)	0.0620 (8)	0.0335 (5)	−0.0121 (5)	0.0123 (4)	−0.0051 (5)
O11	0.0376 (6)	0.0699 (8)	0.0255 (5)	−0.0032 (5)	0.0082 (4)	−0.0126 (5)
N11	0.0322 (6)	0.0450 (8)	0.0292 (6)	−0.0065 (5)	0.0065 (5)	−0.0037 (5)
N1	0.0540 (9)	0.0426 (8)	0.0383 (7)	0.0039 (7)	0.0255 (7)	0.0003 (6)
O1	0.0556 (7)	0.0316 (6)	0.0503 (7)	0.0030 (5)	0.0106 (6)	0.0020 (5)
O2	0.0892 (11)	0.0701 (10)	0.0720 (10)	0.0418 (9)	0.0327 (8)	0.0276 (8)
O3	0.0938 (12)	0.0904 (12)	0.0750 (11)	−0.0459 (10)	0.0344 (9)	−0.0309 (9)
O5	0.0318 (5)	0.0577 (7)	0.0245 (5)	0.0002 (5)	0.0069 (4)	−0.0042 (5)
O4	0.0312 (5)	0.0455 (6)	0.0240 (5)	0.0041 (5)	0.0054 (4)	0.0039 (4)
C15	0.0459 (9)	0.0362 (8)	0.0336 (8)	−0.0085 (7)	0.0173 (7)	−0.0046 (6)
C12	0.0309 (7)	0.0318 (7)	0.0262 (7)	−0.0033 (6)	0.0067 (6)	−0.0024 (5)
C16	0.0328 (8)	0.0505 (10)	0.0389 (8)	−0.0080 (7)	0.0121 (6)	−0.0050 (7)
C13	0.0337 (8)	0.0477 (9)	0.0271 (7)	−0.0078 (7)	0.0071 (6)	−0.0064 (6)
C17	0.0322 (7)	0.0344 (8)	0.0246 (6)	0.0009 (6)	0.0065 (5)	−0.0014 (5)
N12	0.0458 (8)	0.0499 (8)	0.0257 (6)	−0.0101 (6)	0.0090 (5)	−0.0059 (5)
C18	0.0676 (12)	0.0582 (11)	0.0439 (9)	−0.0142 (9)	0.0322 (9)	−0.0076 (8)

### Geometric parameters (Å, °)

**Table:**

Li1—O11^i^	2.029 (3)	C38—H38A	0.9600
Li1—O11	2.039 (3)	C38—H38B	0.9600
Li1—O1	2.085 (3)	C38—H38C	0.9600
Li1—O4	2.114 (3)	O12—C17	1.2397 (18)
Li1—N11	2.293 (3)	O12—Li2^i^	1.958 (3)
Li1—Li1^i^	3.134 (5)	O11—C17	1.2460 (18)
Li2—O12^i^	1.958 (3)	O11—Li1^i^	2.029 (3)
Li2—O4	1.965 (3)	N11—C16	1.328 (2)
Li2—O5	1.970 (3)	N11—C12	1.3404 (19)
Li2—O2^ii^	2.163 (3)	N1—O2	1.219 (2)
Li2—O3^ii^	2.550 (4)	N1—O3	1.225 (2)
Li2—C17^i^	2.677 (3)	N1—O1	1.2516 (19)
Li2—N1^ii^	2.715 (3)	N1—Li2^iii^	2.715 (3)
Li2—H52	2.34 (2)	O2—Li2^iii^	2.163 (3)
Li3—O31	1.978 (3)	O3—Li2^iii^	2.550 (4)
Li3—O5	2.025 (4)	O5—H51	0.86 (2)
Li3—O1	2.039 (3)	O5—H52	0.84 (2)
Li3—N31	2.117 (3)	O4—H42	0.85 (2)
Li3—O2	2.637 (4)	O4—H41	0.85 (2)
O31—C37	1.2573 (19)	C15—N12	1.334 (2)
O32—C37	1.2351 (19)	C15—C16	1.394 (2)
N31—C36	1.328 (2)	C15—C18	1.497 (2)
N31—C32	1.3419 (19)	C12—C13	1.381 (2)
C32—C33	1.384 (2)	C12—C17	1.5134 (18)
C32—C37	1.5205 (19)	C16—H16	0.9300
C33—N32	1.332 (2)	C13—N12	1.336 (2)
C33—H33	0.9300	C13—H13	0.9300
N32—C35	1.334 (2)	C17—Li2^i^	2.677 (3)
C35—C36	1.392 (2)	C18—H18A	0.9600
C35—C38	1.498 (2)	C18—H18B	0.9600
C36—H36	0.9300	C18—H18C	0.9600
			
O11^i^—Li1—O11	79.21 (10)	N31—C36—H36	118.6
O11^i^—Li1—O1	99.02 (11)	C35—C36—H36	118.6
O11—Li1—O1	132.82 (15)	O32—C37—O31	127.21 (13)
O11^i^—Li1—O4	95.45 (12)	O32—C37—C32	117.25 (13)
O11—Li1—O4	137.34 (15)	O31—C37—C32	115.53 (13)
O1—Li1—O4	89.83 (11)	C35—C38—H38A	109.5
O11^i^—Li1—N11	148.03 (14)	C35—C38—H38B	109.5
O11—Li1—N11	75.84 (9)	H38A—C38—H38B	109.5
O1—Li1—N11	112.56 (12)	C35—C38—H38C	109.5
O4—Li1—N11	89.63 (10)	H38A—C38—H38C	109.5
O11^i^—Li1—Li1^i^	39.72 (7)	H38B—C38—H38C	109.5
O11—Li1—Li1^i^	39.50 (7)	C17—O12—Li2^i^	111.69 (12)
O1—Li1—Li1^i^	122.93 (15)	C17—O11—Li1^i^	133.79 (12)
O4—Li1—Li1^i^	122.68 (16)	C17—O11—Li1	121.84 (11)
N11—Li1—Li1^i^	112.96 (13)	Li1^i^—O11—Li1	100.79 (10)
O12^i^—Li2—O4	124.82 (15)	C16—N11—C12	116.54 (13)
O12^i^—Li2—O5	100.73 (13)	C16—N11—Li1	133.87 (12)
O4—Li2—O5	96.72 (12)	C12—N11—Li1	108.90 (11)
O12^i^—Li2—O2^ii^	105.34 (13)	O2—N1—O3	119.92 (19)
O4—Li2—O2^ii^	123.98 (14)	O2—N1—O1	119.79 (17)
O5—Li2—O2^ii^	96.79 (12)	O3—N1—O1	120.29 (17)
O12^i^—Li2—O3^ii^	109.12 (12)	O2—N1—Li2^iii^	50.83 (12)
O4—Li2—O3^ii^	85.96 (11)	O3—N1—Li2^iii^	69.11 (13)
O5—Li2—O3^ii^	141.47 (14)	O1—N1—Li2^iii^	170.49 (13)
O2^ii^—Li2—O3^ii^	52.56 (8)	N1—O1—Li3	107.95 (14)
O12^i^—Li2—C17^i^	25.49 (5)	N1—O1—Li1	114.41 (13)
O4—Li2—C17^i^	101.89 (11)	Li3—O1—Li1	137.29 (14)
O5—Li2—C17^i^	117.81 (13)	N1—O2—Li2^iii^	103.26 (16)
O2^ii^—Li2—C17^i^	118.44 (11)	N1—O2—Li3	79.75 (13)
O3^ii^—Li2—C17^i^	98.94 (10)	Li2^iii^—O2—Li3	176.60 (12)
O12^i^—Li2—N1^ii^	109.68 (12)	N1—O3—Li2^iii^	84.22 (14)
O4—Li2—N1^ii^	105.79 (12)	Li2—O5—Li3	109.33 (13)
O5—Li2—N1^ii^	119.60 (13)	Li2—O5—H51	117.3 (14)
O2^ii^—Li2—N1^ii^	25.90 (6)	Li3—O5—H51	109.4 (14)
O3^ii^—Li2—N1^ii^	26.66 (5)	Li2—O5—H52	105.8 (15)
C17^i^—Li2—N1^ii^	111.17 (10)	Li3—O5—H52	111.6 (15)
O12^i^—Li2—H52	94.5 (6)	H51—O5—H52	103 (2)
O4—Li2—H52	115.4 (6)	Li2—O4—Li1	99.79 (11)
O5—Li2—H52	20.1 (6)	Li2—O4—H42	119.7 (14)
O2^ii^—Li2—H52	80.0 (6)	Li1—O4—H42	105.3 (14)
O3^ii^—Li2—H52	130.7 (6)	Li2—O4—H41	109.1 (14)
C17^i^—Li2—H52	117.2 (6)	Li1—O4—H41	119.0 (14)
N1^ii^—Li2—H52	105.0 (6)	H42—O4—H41	104.7 (19)
O31—Li3—O5	97.02 (14)	N12—C15—C16	119.77 (13)
O31—Li3—O1	141.39 (18)	N12—C15—C18	117.61 (15)
O5—Li3—O1	100.53 (13)	C16—C15—C18	122.62 (15)
O31—Li3—N31	81.64 (11)	N11—C12—C13	120.98 (13)
O5—Li3—N31	110.61 (16)	N11—C12—C17	116.84 (12)
O1—Li3—N31	122.54 (15)	C13—C12—C17	122.18 (13)
O31—Li3—O2	99.10 (14)	N11—C16—C15	123.00 (14)
O5—Li3—O2	150.13 (14)	N11—C16—H16	118.5
O1—Li3—O2	52.51 (9)	C15—C16—H16	118.5
N31—Li3—O2	96.54 (13)	N12—C13—C12	122.10 (14)
C37—O31—Li3	115.81 (12)	N12—C13—H13	119.0
C36—N31—C32	116.92 (13)	C12—C13—H13	119.0
C36—N31—Li3	135.00 (13)	O12—C17—O11	126.37 (13)
C32—N31—Li3	108.08 (12)	O12—C17—C12	117.84 (12)
N31—C32—C33	120.58 (13)	O11—C17—C12	115.79 (13)
N31—C32—C37	116.98 (12)	O12—C17—Li2^i^	42.82 (9)
C33—C32—C37	122.43 (13)	O11—C17—Li2^i^	85.43 (10)
N32—C33—C32	122.04 (14)	C12—C17—Li2^i^	155.18 (12)
N32—C33—H33	119.0	C15—N12—C13	117.58 (13)
C32—C33—H33	119.0	C15—C18—H18A	109.5
C33—N32—C35	117.83 (13)	C15—C18—H18B	109.5
N32—C35—C36	119.78 (14)	H18A—C18—H18B	109.5
N32—C35—C38	117.75 (15)	C15—C18—H18C	109.5
C36—C35—C38	122.47 (15)	H18A—C18—H18C	109.5
N31—C36—C35	122.80 (14)	H18B—C18—H18C	109.5
			
O5—Li3—O31—C37	−97.15 (15)	O11^i^—Li1—O1—Li3	59.4 (2)
O1—Li3—O31—C37	146.2 (2)	O11—Li1—O1—Li3	142.98 (18)
N31—Li3—O31—C37	12.75 (18)	O4—Li1—O1—Li3	−36.13 (18)
O2—Li3—O31—C37	108.09 (15)	N11—Li1—O1—Li3	−125.66 (17)
O31—Li3—N31—C36	174.23 (18)	Li1^i^—Li1—O1—Li3	93.8 (2)
O5—Li3—N31—C36	−91.4 (2)	O3—N1—O2—Li2^iii^	−1.90 (17)
O1—Li3—N31—C36	26.8 (3)	O1—N1—O2—Li2^iii^	178.05 (12)
O2—Li3—N31—C36	75.9 (2)	O3—N1—O2—Li3	179.73 (15)
O31—Li3—N31—C32	−6.47 (16)	O1—N1—O2—Li3	−0.32 (13)
O5—Li3—N31—C32	87.93 (16)	Li2^iii^—N1—O2—Li3	−178.38 (12)
O1—Li3—N31—C32	−153.94 (18)	O31—Li3—O2—N1	151.21 (13)
O2—Li3—N31—C32	−104.75 (13)	O5—Li3—O2—N1	29.4 (3)
C36—N31—C32—C33	−1.3 (2)	O1—Li3—O2—N1	0.22 (9)
Li3—N31—C32—C33	179.29 (17)	N31—Li3—O2—N1	−126.25 (13)
C36—N31—C32—C37	−179.90 (15)	O31—Li3—O2—Li2^iii^	−1(2)
Li3—N31—C32—C37	0.65 (19)	O5—Li3—O2—Li2^iii^	−122.9 (18)
N31—C32—C33—N32	−0.8 (3)	O1—Li3—O2—Li2^iii^	−152.1 (19)
C37—C32—C33—N32	177.77 (15)	N31—Li3—O2—Li2^iii^	81.4 (19)
C32—C33—N32—C35	2.2 (3)	O2—N1—O3—Li2^iii^	1.58 (14)
C33—N32—C35—C36	−1.5 (2)	O1—N1—O3—Li2^iii^	−178.38 (13)
C33—N32—C35—C38	178.88 (17)	O12^i^—Li2—O5—Li3	−46.98 (16)
C32—N31—C36—C35	1.9 (3)	O4—Li2—O5—Li3	80.43 (14)
Li3—N31—C36—C35	−178.82 (18)	O2^ii^—Li2—O5—Li3	−154.06 (12)
N32—C35—C36—N31	−0.5 (3)	O3^ii^—Li2—O5—Li3	172.38 (19)
C38—C35—C36—N31	179.02 (17)	C17^i^—Li2—O5—Li3	−26.82 (17)
Li3—O31—C37—O32	164.44 (18)	N1^ii^—Li2—O5—Li3	−167.10 (12)
Li3—O31—C37—C32	−15.7 (2)	O31—Li3—O5—Li2	176.72 (12)
N31—C32—C37—O32	−170.32 (15)	O1—Li3—O5—Li2	−37.82 (15)
C33—C32—C37—O32	11.1 (2)	N31—Li3—O5—Li2	93.04 (15)
N31—C32—C37—O31	9.8 (2)	O2—Li3—O5—Li2	−61.0 (3)
C33—C32—C37—O31	−168.76 (16)	O12^i^—Li2—O4—Li1	7.0 (2)
O11^i^—Li1—O11—C17	−161.36 (16)	O5—Li2—O4—Li1	−101.06 (12)
O1—Li1—O11—C17	106.3 (2)	O2^ii^—Li2—O4—Li1	156.06 (15)
O4—Li1—O11—C17	−75.0 (2)	O3^ii^—Li2—O4—Li1	117.56 (10)
N11—Li1—O11—C17	−1.51 (16)	C17^i^—Li2—O4—Li1	19.27 (13)
Li1^i^—Li1—O11—C17	−161.36 (16)	N1^ii^—Li2—O4—Li1	135.57 (11)
O11^i^—Li1—O11—Li1^i^	0.0	O11^i^—Li1—O4—Li2	−32.20 (13)
O1—Li1—O11—Li1^i^	−92.35 (18)	O11—Li1—O4—Li2	−112.19 (19)
O4—Li1—O11—Li1^i^	86.3 (2)	O1—Li1—O4—Li2	66.84 (12)
N11—Li1—O11—Li1^i^	159.85 (14)	N11—Li1—O4—Li2	179.41 (11)
O11^i^—Li1—N11—C16	−134.3 (3)	Li1^i^—Li1—O4—Li2	−63.25 (18)
O11—Li1—N11—C16	−174.07 (17)	C16—N11—C12—C13	1.3 (2)
O1—Li1—N11—C16	55.1 (2)	Li1—N11—C12—C13	−170.60 (14)
O4—Li1—N11—C16	−34.6 (2)	C16—N11—C12—C17	−179.63 (14)
Li1^i^—Li1—N11—C16	−160.31 (17)	Li1—N11—C12—C17	8.50 (17)
O11^i^—Li1—N11—C12	35.6 (3)	C12—N11—C16—C15	−1.4 (2)
O11—Li1—N11—C12	−4.18 (14)	Li1—N11—C16—C15	167.87 (16)
O1—Li1—N11—C12	−135.04 (14)	N12—C15—C16—N11	0.1 (3)
O4—Li1—N11—C12	135.30 (12)	C18—C15—C16—N11	179.94 (16)
Li1^i^—Li1—N11—C12	9.6 (2)	N11—C12—C13—N12	0.3 (2)
O2—N1—O1—Li3	0.43 (18)	C17—C12—C13—N12	−178.79 (14)
O3—N1—O1—Li3	−179.62 (14)	Li2^i^—O12—C17—O11	19.9 (2)
Li2^iii^—N1—O1—Li3	9.6 (7)	Li2^i^—O12—C17—C12	−160.09 (13)
O2—N1—O1—Li1	174.88 (13)	Li1^i^—O11—C17—O12	32.3 (3)
O3—N1—O1—Li1	−5.17 (19)	Li1—O11—C17—O12	−173.52 (16)
Li2^iii^—N1—O1—Li1	−175.9 (6)	Li1^i^—O11—C17—C12	−147.73 (16)
O31—Li3—O1—N1	−50.3 (3)	Li1—O11—C17—C12	6.5 (2)
O5—Li3—O1—N1	−165.93 (12)	Li1^i^—O11—C17—Li2^i^	45.70 (19)
N31—Li3—O1—N1	71.2 (2)	Li1—O11—C17—Li2^i^	−160.08 (14)
O2—Li3—O1—N1	−0.22 (9)	N11—C12—C17—O12	169.59 (14)
O31—Li3—O1—Li1	137.1 (2)	C13—C12—C17—O12	−11.3 (2)
O5—Li3—O1—Li1	21.5 (2)	N11—C12—C17—O11	−10.4 (2)
N31—Li3—O1—Li1	−101.4 (2)	C13—C12—C17—O11	168.67 (15)
O2—Li3—O1—Li1	−172.75 (16)	N11—C12—C17—Li2^i^	136.1 (2)
O11^i^—Li1—O1—N1	−112.83 (13)	C13—C12—C17—Li2^i^	−44.8 (3)
O11—Li1—O1—N1	−29.2 (2)	C16—C15—N12—C13	1.5 (2)
O4—Li1—O1—N1	151.67 (11)	C18—C15—N12—C13	−178.41 (16)
N11—Li1—O1—N1	62.14 (16)	C12—C13—N12—C15	−1.7 (2)
Li1^i^—Li1—O1—N1	−78.4 (2)		

Symmetry codes: (i) −*x*+2, −*y*, −*z*+2; (ii) *x*, *y*−1, *z*; (iii) *x*, *y*+1, *z*.

### Hydrogen-bond geometry (Å, °)

**Table:**

*D*—H···*A*	*D*—H	H···*A*	*D*···*A*	*D*—H···*A*
O4—H41···O32^iv^	0.85 (2)	1.92 (2)	2.7449 (15)	165.3 (19)
O4—H42···N12^v^	0.85 (2)	2.03 (2)	2.8414 (17)	159.0 (19)
O5—H52···N32^vi^	0.84 (2)	2.05 (2)	2.8550 (17)	162 (2)
O5—H51···O31^iv^	0.86 (2)	1.85 (2)	2.7055 (15)	172 (2)

Symmetry codes: (iv) −*x*+1, −*y*, −*z*+2; (v) −*x*+2, *y*−1/2, −*z*+5/2; (vi) −*x*+1, *y*−1/2, −*z*+3/2.
